# Self-perception of health and physical activity levels among the youth and adults before and amidst the COVID-19 pandemic

**DOI:** 10.3389/fpubh.2024.1298378

**Published:** 2024-05-30

**Authors:** Vida Korleki Nyawornota, Clement Adamba, Doris Akosua Tay, Oscar C. Nyanyofio, Rosemary C. Muomah, JohnBosco C. Chukwuorji, Sampson K. Nwonyi, Leapetswe Malete, Dale Joachim, Reginald T. Ocansey

**Affiliations:** ^1^Department of Physical Education and Sport Studies, University of Ghana, Accra, Ghana; ^2^Department of Educational Studies and Leadership, University of Ghana, Accra, Ghana; ^3^Regional Institute for Population Studies, University of Ghana, Accra, Ghana; ^4^Department of Psychological Medicine, University of Nigeria, Nsukka, Nigeria; ^5^Charles Stewart Mott Department of Public Health, Michigan State University, East Lansing, MI, United States; ^6^Department of Psychology, University of Nigeria, Nsukka, Nigeria; ^7^Department of Psychology, Ebonyi State University, Abakaliki, Nigeria; ^8^Department of Kinesiology, Michigan State University, East Lansing, MI, United States; ^9^Sonde Health, Boston, MA, United States

**Keywords:** COVID-19, Ghana, physical activity, health status, gender, public health

## Abstract

**Background:**

Emerging research indicates that the COVID-19 pandemic and associated restrictions led to decreased physical activity levels and poorer health globally. However, most studies on this topic have focused on advanced countries, leaving a gap in understanding the impact in countries like Ghana. This study aimed to fill this gap by assessing self-perceived health status and physical activity levels among youth and adults in Ghana before, during, and after the COVID-19 restrictions. Understanding these dynamics is crucial for informing public health interventions and policies to promote well-being during and beyond the pandemic.

**Methods:**

A cross-sectional survey using online data collection methods was conducted, involving 937 participants. Data included demographic information, and International Physical Activity Questionnaire-Short Form (IPAQ-SF). Analysis was done using SPSS version 25, with descriptive statistics and multinomial regression.

**Results:**

Most participants (89.6%) reported good health. Male participants were significantly more likely to engage in moderate (1.78 times) and high (3.17 times) physical activity during the COVID-19 period compared to females.

**Conclusion:**

This study highlights gender disparities in physical activity levels during the pandemic in Ghana. Addressing these disparities and promoting healthier lifestyles, especially during crises, is crucial for general and mental health. Further research should explore socio-demographic factors’ role in health behaviors during pandemics.

## Introduction

The Coronavirus (COVID-19) pandemic has been a global health crisis since its emergence in December 2019 (1–9). With its highly infectious nature, the virus quickly spread worldwide, posing significant public health risks (1, 10–16). The ensuing restrictions implemented to curb its spread have profoundly impacted various aspects of society, including restricting access to physical activity opportunities (15, 17–19). As people navigated movement limitations, lockdowns, and the socio-economic repercussions of COVID-19, overall health and well-being were significantly affected (4, 8, 10, 20–23). The pandemic introduced new stressors and disruptions to daily life, heightening concerns about personal health and welfare (16, 24). Recognizing the multidimensional nature of health, the World Health Organization advocates for the use of self-reported health measures to assess perceived health status (25). This approach considers physical, mental, and social dimensions of health, influenced by factors such as socio-economic status and lifestyle (14, 26).

Evidence from research conducted in Ghana highlights the importance of self-perceived health assessments within the population. Previous studies have investigated various predictors of self-rated health, including cultural factors, age, and income (27, 28). These studies have shown that perceived health status is closely linked to functional decline, with poorer self-rated health associated with declines in functionality (29). Additionally, associations have been found between functional and general health literacy and self-rated health status, particularly among vulnerable populations such as street youth (30). Young adults in Ghana have been observed to exhibit more positive self-perceptions of health compared to older adults, emphasizing the role of age in health perception (31). Furthermore, predictors of self-rated health among older adults in Ghana have been explored, revealing associations with factors such as age, income, and tobacco use (27).

Evidence also suggests that individuals’ perception of their environment can influence their self-perceived health status (32). Negative perceptions of the environment have been associated with poorer self-rated (32). In sub-Saharan Africa, studies on self-perception of health have been conducted, providing insights into health status among different (33, 34). For example, a study in Togo found that self-rated health status was generally poor among older adults, particularly females, with factors such as osteoarthritis and polypharmacy being associated with poor health status (33). In Burkina Faso, the prevalence of self-rated poor health was lower, with associations found with age, marital status, and chronic diseases (34).

Regular physical activity (PA) has been associated with numerous health benefits, including reducing the risk of mood disorders such as depression and anxiety, as well as improving heart health (1, 10, 35). Studies have consistently shown that moderate to high-intensity exercise is particularly effective in lowering levels of depression and enhancing overall well-being (1, 10, 36–39). However, the COVID-19 pandemic and its associated restrictions, including lockdowns, quarantines, and travel limitations, have likely had a negative impact on regular PA and exercise routines. These restrictions have limited opportunities for physical activity and may have contributed to increased sedentary behavior, such as prolonged screen time due to stay-at-home measures and online learning (1, 2, 8, 19, 40–43).

Despite research conducted on self-perception of health, little is known about the relationship between self-perception of health and physical activity during the COVID-19 pandemic in Ghana (39, 44). Therefore, this study aimed to assess the perceived health status and physical activity behavior of Ghanaian youth and adults before, during, and after the COVID-19 pandemic restrictions. Concerns have been raised regarding the significant impact of the COVID-19 pandemic on the health of populations globally (1, 39, 44). This impact could be particularly severe on African populations due to under-resourced health systems, potentially influencing their perception of health (24, 44, 45).

## Methods

This study is part of a multi-country project including Ghana, Nigeria, Tanzania, and Botswana. The multi-country project focused on health, speech, and PA relationship assessment in the general sense. However, this paper focused specifically on PA, and self-rated health status among participants in the data germane to the period during the COVID-19 pandemic within the Ghana context.

### Study participants

The participants were included if they were 18 years of age and above. People who were below 18 years were excluded from the study.

### Ethics statement

The study was approved by the Ethical Committees for Humanities (ECH) of the University of Ghana, with clearance number ECH162/19-20 given on 5 June 2020 and valid till 4th August 2023. The study was designed to assess the effects of COVID-19 pandemic, on physical activity and general health. The recruitment of participants was in two phases. Phase one was carried out between April 2021 and September 2021 and was online. The second phase took place within 22 August 2021 and 26 August 2022, and the participants were approached on-site recruitment at University of Ghana. The participants were recruited through email invitations (University of Ghana Mail), social media platforms (WhatsApp, Facebook), snowballing, and through face-to-face. Before the second phase began, four research assistants were recruited and trained to assist with data collection. This was mostly through face-to-face recruitment at the University of Ghana. The data was stored on secure HIPAA (Health Insurance Portability and Accountability Act) compliant Amazon AWS (Amazon Web Services) server, fully anonymized, and was accessible to the project team members. The data was used for the purpose it was collected only.

### Data collection

An online survey lasting 30 min was employed to collect data germane to physical activity participation and perceived health status. Participants consented to being contacted in the future to complete the survey a second time. The participants were compensated with data bundle worth GHC 10.

The SurveyLex web-based platform was used for collecting data for this study, and was developed by Sonde Health, Michigan State University, and the Principal Investigators from Ghana, Botswana, Nigeria, and Tanzania. The tools used for data collection for this study, included the IPAQ-SF (International Physical Activity Questionnaire-Short form). This was utilized to assess the participants’ level of PA participation. Scoring was based on the total duration of PA per week recommended by the WHO. A general health status questionnaire was also employed to gauge the perception of health of the participants. These tools were approved by the during ethical clearance.

The datasets were cleaned to exclude duplicates, incomplete responses, and participants who indicated their ages were below 18 years.

### Measures

Participants completed a form that assessed their demographic characteristics as well as self-rated health status, and PA engagement. Participants were required to declare their health status as either poor, fair, good, very good, and excellent. Participants were allowed to select an option ‘do not know’ if they did not have an answer. The categories of self-rated health status were recoded into two variables namely poor (includes poor, fair, do not know) and good (includes good, very good, and excellent) health status due to sparse distribution and to aid in analysis.

The IPAQ-SF is a seven-item self-report measure of PA and inactivity. It was used to collect information on the number of days and time spent on PA with vigorous intensity, moderate intensity, walking for at least 10 mins at a time in the last 7 days as well as time spent sitting on a weekday. Respondents were asked to indicate the number of days per week, hours, and minutes per day they spent doing PA within the categories. They may also indicate that they were not sure of the activity undertaken. These activity categories were treated separately to obtain activity levels including low activity, moderate activity or high activity which were interpreted as below, meeting, or exceeding recommendations. The Cronbach’s alpha for the IPAQ items in this study was 0.84.

## Results

The characteristics of respondents, places for physical activity and self-perceptions of health status of all the 937 participants are displayed in Table 1. The participants included 56.5% males and 45% females. The 20–24 years age-group constituted the highest group of participants in the study, with a mean age of 23.6%. More than 50% of the participants have tertiary education. As many as 80% of participants are students, only about 13% of the study participants have formal employment. Fifty-eight percent (58%) of respondent have not been vaccinated against the COVID-19 pandemic as at the time of the study. Most participants perceived their health status to be excellent and very good conditions. Responding to place where participants performed physical activity, about 30% indicated no physical activity whereas almost half of them said they performed PA at home.

**Table 1:** Characteristics of respondents, places of physical activity, and perceived health status.

Items	Percent (%)
**Gender**	
Female	43.5
Male	56.5
**Age group**	
18–19 years	13.4
20-24 years	60.6
25-29 years	16.8
30-34 years	4.4
35-39yeasr	2.1
40 years+	2.7
**Education level**	
Junior High School and below	6.9
Secondary/Technical/Vocational	37.8
Tertiary	55.0
**Employment**	
Formal Employment	12.9
Retired	0.1
Self-employed	2.9
Student	80.0
Unemployed	3.6
**COVID-19 vaccination**	
No	58.0
Yes	41.9
**Perceived health status**	
Excellent	23.6
Very good	35.4
Good	22.8
Fair	7.7
Poor	10.4
**Weight**	
Normal	80.5
Underweight	7.6
Overweight	9.5
Obese	1.5
**Place of physical activity**	
No PA	30.5
Home	49.3
Neighborhood	11.6
Town	6.2
Grocery shop	2.3

**Figure 1:**
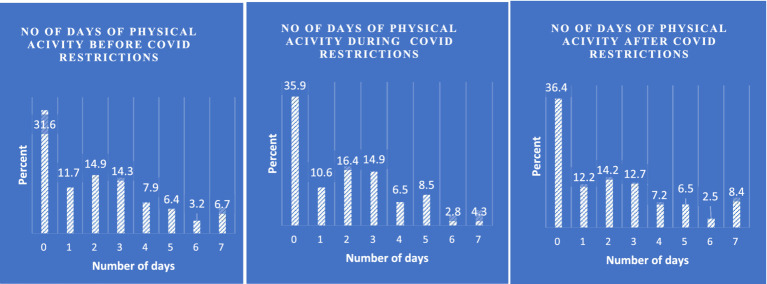
Number of days of physical activity before, during and after the COVID-19 pandemic restriction in Ghana.

Figure 1 shows the frequency of physical activity among respondents before, during and after the COVID-19 pandemic restrictions in Ghana. Proportion of participant not engaging in any PA activity throughout the week before the COVID-19 pandemic was about 31%, the number increased during the COVID-19 restriction (35.9%) and after the restriction (36%). Also, the proportion of participants engaged in PA throughout the week before the restriction was 6.7%, this dropped to 4.3% during the restriction and then increased to 8.4% after the restriction. More people were physically inactive during the COVID-19 restriction, and this may be due to fear and the restriction on movement and the lockdown. Participants reporting 2 and 3 days of PA rather have increasing numbers during the COVID-19 restriction.

**Figure 2:**
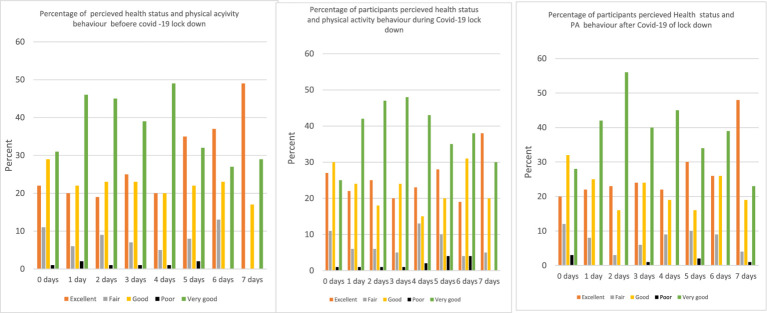
Participants perceived health status and frequency of physical activity per week before during and after COVID-19 restriction in Ghana.

Figure 2 shows the frequency of participants PA behavior per week, and perceived heath status, before, during and after the COVID-19 pandemic restriction. Irrespective of the frequency of PA per week, most participants perceived their health status to be good, very good and excellent before, during and after the restriction, with a little below 10% reporting poor health status. However, those who perceived their health status to be poor slightly increased during the restriction periods among the 5-days and 6-days PA activity groups. Participants engaging in PA for 6 and 7 days did not perceive their health status as poor before the pandemic restriction, some of them in the 6 days category did during the lockdown, and even after the restriction among those doing PA all 7 days in the week.

During the COVID-19 restriction almost half of the total number of participants in the study reported engaging in PA at home as shown in Figure 2, and this may be as a result of the lockdown and the fear of contacting the disease when they go out.

Table 2 portrays the perceived health status of participants in the study. More male participants perceived their health status to be good, very good and excellent compared to their female counterparts during the COVID-19 restrictions in Ghana. While 74% of males indicted that their health status was excellent, only 23% of the female counterpart said their health status was excellent. Participants within 20–24 years of age perceived their health status as good, very good and excellent. Participants who engaged in high level PA had 50% who perceived their health status to be excellent, whereas those who engaged in low PA had 31% also perceived their health status as excellent during the pandemic restriction.

**Table 2:** Percentage distribution of characteristics, health status, and PA participation in Ghana during COVID-19 pandemic restriction.

	Excellent	Very good	Good	Fair	Poor	Do not know
**Gender**						
Female	16	37	30	11	3	3
Male	31	39	20	7	0	2
**Age-group**						
18–19 years	14	11	11	17	55	16
20–24 years	60	6	65	57	27	68
25–30 years	16	21	13	13	9	16
31–34 years	6	4	4	4	0	0
35–40yeasr	3	2	2	0	0	0
41 years+	1	3	4	3	1	0
**PA levels**						
Low PA	31	26	37	37	80	30
Moderate PA	19	34	29	31	0	35
High PA	50	40	34	32	20	35
**Employment**						
Formal employment	16	1	16	4	9	5
Self-employed	2	14	3	1	0	10
Student	79	3	74	85	91	76
Unemployed	3	81	6	10	0	10
Retired/Pensioner	0	1	0	0	0	0
**Education**						
JHS	0	0	0	0	1	0
Secondary/Tech/Voc school	58	37	82	42	33	3
Tertiary	33	48	2	50	61	48
**COVID-19 vaccination**						
No	54	53	58	61	64	67
Yes	45	47	42	39	36	29

Table 3 displays a multinomial regression analysis of the relationship between participants demographics, perceived health status, and physical activity levels during COVID-19 restriction in Ghana. Prior to the logistic regression analysis, correlation diagnostics were performed to ensure that high inter-correlations among the predictor variables were avoided. The Spearman’s correlation coefficients were less than 0.7 for all included variables which suggested no multicollinearity existed among the independent variables.

**Table 3:** Multinomial regression analysis of relationship between participants demographic and perceived health status with physical activity levels during COVID-19 restriction in Ghana.

	Physical activity			
	Model A (odds ratio, 95 CI)			
Variables	Moderate	*p*-value	High	*P*-value
**Gender**				
Male	1.788 (1.243, 2.572)	0.002	3.174 (2.223, 4.534)	0.000
Female	Reference			
**Age-groups**				
18–30 years	1.098 (0.655, 1.840)	0.724	1.162 (0.706, 1.914)	0.555
31 years+	Reference			
**Place of PA**				
No PA	0.325(0.185, 0.569)	0.000	0.162 (0.097, 0.270)	0.000
PA at home	1.038(0.622, 1.732)	0.887	0.513 (0.323, 0.815)	0.005
Neighborhood	Reference			
**Perceived health status**				
Very good	1.050(0.620, 1.777)	0.856	1.188 (0.712, 1.983)	0.510
Good	0.902(0.505, 1.611)	0.727	0.812 (0.458, 1.439)	0.475
Poor	Reference			
**COVID-19 vaccination**				
No	0.841(0.586, 1.2030)	0.344	771(0.546, 1.087)	0.138
Yes	Reference			

Variables with a statistically significant association (*p* < 0.05) and variables that trended toward significance (0.05 ≤ *p* ≤ 0.10) with the likelihood of moderate/high PA on a univariate analysis were the only variables analyzed using logistic regression modeling. Low physical activity was the reference category compared to moderate and high PA.

Also, for each of the variables, the last variable option was used as the reference for comparison. The model explains between 14.4% (Cox and Snell R2) and 16.2% (Nagelkerke R2) of the variance.

The results are presented in adjusted odds ratios with 95% confidence intervals. Significant was set at 0.05 alpha level. Physical activity levels (Low PA, Moderate PA and High PA) are the dependent variable.

As indicated in Table 3, gender showed a positive significant association with moderate (*p* = 0.002) and high (*p* = 0.000) physical activity. Being a male was significantly associated with engaging in PA during the COVID-19 period in Ghana. Male participants were 1.78 times and 3.17 times more likely to engage in moderate and high PA, respectively, during the COVID-19 period. Age of participants, and COVID-19 vaccination were not significantly associated with PA levels during the COVID-19 period in this study. Education level, employment status and weight of participants were not included in the final modeling because they did not show significance in the prediction modeling stage.

Places where participants engaged in PA during the COVID-19 pandemic showed significant association with levels of PA. Participant who responded ‘No PA’ showed negative significant association with moderate (*p* = 0.000) and high (*p* = 0.000) PA. Engaging in PA at home during the COVID-19 pandemic also showed negative significant association with high physical activity (*p* = 0.005). Participants who did not do any PA anywhere during the COVID-19 pandemic were about 33% less likely to have moderate level PA, and those at home are 103% more likely to have moderate physical activity levels, compared to those engaging in physical activity in their neighborhood. Those who did PA at home were also 51% less likely to have high PA levels than those doing PA in their neighborhood. The COVID-19 period was characterized with home PA and this may be due to fear of contacting the disease, the lockdown and restriction, and the closing down of shops and recreational facilities. The fear of COVID-19 and the restriction may have confined people to doing physical activity in their homes than moving out to the neighborhood to engage in any physical activity.

The place of residence of participants was not significantly associated with PA levels. Where participant resides, be it national, capital, town or village did not influence PA behavior in this study. Although how participants perceived their health status during the COVID-19 pandemic were not significantly associated with their levels of PA, those who perceived their health status to be very good and good were 105% more likely, and 90% less likely respectively, to have moderate PA compared to those with poor health perceptions in this study. Also, participants perceiving their health status as very good and good were about 119% more likely and 81% less likely to have high levels of physical activity, respectively, compared to those with poor health status during the COVID-19 pandemic.

## Discussion

The results of this study showed slight variation in PA behavior among participants before, during and after the COVID-19 pandemic restrictions. The study reported a reduction in PA levels during the pandemic restriction, which is consistent with other studies on the impact of COVID-19 and PA behavior that also reported a reduction in PA levels (1, 35, 46). The study also found that more males were engaging in PA than their female counterparts, which is consistent with studies on COVID-19, health, and PA behavior (1, 13, 35, 47). Additionally, participants engaging in PA all the days of the week did not perceive their health status as poor before the COVID-19 restrictions.

Half of the study participants reported performing physical activity at home, which is reflective of findings from other studies. Dunton et al. reported that during the COVID-19 pandemic, a greater proportion (75%) of PA was reported at home than on sidewalks and/or roads outside in the neighborhood, which is consistent with the finding from this study as well findings of related research (35, 48, 49). Performing PA at home was significantly associated with high or vigorous levels of PA even after adjusting for age and sex in this study, which supports other studies conducted during the COVID-19 pandemic (35, 48). The home became the safest place to perform PA during the COVID-19 period due to fear of contact with the diseases, movement restrictions, closure of businesses and lockdowns in Ghana, like other countries that experienced pandemic restrictions.

Furthermore, a greater number of male participants in this study perceived their health status to be excellent and very good than the females in the study, which is also reflective of findings from other studies (1, 13, 47, 50). COVID-19 restrictions affected the health status of females more than males. Hence, it is important to consider gender-based approaches to interventions that would support wellbeing during pandemics.

## Conclusion

Although the COVID-19 pandemic retractions affected some health behaviors among participants, including a decline in the frequency of weekly PA behavior, study participants who engaged in PA were more likely to perceive their health status to be excellent and particularly good. Individuals who take part in PA were likely to have improvements in their physical, mental, and emotional wellbeing. Therefore, people should get regular exercises because it not only improves cardiovascular and general physical health, but also mood, focus, and mental alertness. This study recorded more participants performing PA in their homes during the pandemic. The home was a significant place for people to engage in moderate to vigorous PA for improved health during the pandemic restriction. It is recommended that during such pandemic situations that are accompanied by movement restrictions and lockdowns, there should be strong advocacy for people to engage in physical activities in their various homes to help maintain good physical and mental health.

## Data availability statement

The raw data supporting the conclusions of this article will be made available by the authors, without undue reservation.

## Ethics statement

The studies involving humans were approved by the University of Ghana, Legon, Ghana. Ethics Committee for the Humanities approval (ECH 162/19-20). The studies were conducted in accordance with the local legislation and institutional requirements. The participants provided their written informed consent to participate in this study.

## Author contributions

VN: Data curation, Formal analysis, Methodology, Validation, Writing – original draft. CA: Conceptualization, Data curation, Methodology, Project administration, Supervision, Validation, Visualization, Writing – review & editing. DT: Data curation, Formal analysis, Methodology, Resources, Writing – original draft. ON: Data curation, Formal analysis, Investigation, Resources, Software, Visualization, Writing – original draft. RM: Methodology, Resources, Visualization, Writing – review & editing. JC: Data curation, Formal analysis, Methodology, Resources, Validation, Visualization, Writing – original draft. SN: Data curation, Formal analysis, Resources, Visualization, Writing – review & editing. LM: Conceptualization, Data curation, Formal analysis, Methodology, Validation, Visualization, Writing – review & editing. DJ: Conceptualization, Data curation, Formal analysis, Methodology, Resources, Software, Validation, Visualization, Writing – review & editing. RO: Conceptualization, Funding acquisition, Project administration, Supervision, Writing – review & editing.
